# Commentary: Reducing Viability Bias in Analysis of Gut Microbiota in Preterm Infants at Risk of NEC and Sepsis

**DOI:** 10.3389/fcimb.2018.00212

**Published:** 2018-06-20

**Authors:** Gemma Agustí, Francesc Codony

**Affiliations:** ^1^Departament d'Òptica i Optometria, Universitat Politècnica de Catalunya-Barcelona Tech, Terrassa, Spain; ^2^Laboratori Municipal - Aigües de Mataró, Mataró, Spain

**Keywords:** viability PCR, live-dead distinction, analysis bias, microbiome, PMA

The recent work by Young et al. (2017) demonstrates the importance of obtaining accurate data when analyzing the microbial community in real-life complex samples. These authors showed that an analysis of gut microbiota samples from preterm infants who at risk of necrotizing enterocolitis (NEC) and sepsis could be biased if the analysis includes DNA from dead cells. The study used a viability PCR (vPCR) approach to overcome this obstacle. Notably, vPCR uses specific photoreactive dye that cannot cross intact cell membranes. When a sample is incubated with this dye and exposed to light, the dye irreversibly binds to DNA in dead cells that have damaged cell membranes. This step effectively neutralizes the DNA and prevents it from being detected by PCR.

Regarding the importance of removing DNA from dead cells during microbial analysis, similar conclusions have been drawn by studies of, for example, environmental samples (Carini et al., 2016) and clinical specimens (Rogers et al., 2013). We agree with this premise and encourage others to use vPCR in microbial analysis, but caution that proper sample preparation is required for this technique.

vPCR methodology is theoretically simple to use since it requires only a reagent and a light source; however, as with many techniques, a lack of practical experience with the technique led the authors to overlook some important considerations. vPCR is still relatively new, with the first paper by Nogva et al. (2003). The scientific community has developed strategies and general rules for optimizing the vPCR workflow; some of these strategies were reviewed by Fittipaldi et al. (2012). More recent work has demonstrated that during sample manipulation, plastic can contribute to false positive results. Indeed, the walls of microtubes can retain free DNA, thereby preventing the nucleic acid from interacting with the reagent. Accordingly, the DNA fraction on the walls of the microtubes is not neutralized, and when it is detected, it is incorrectly assigned to the live DNA fraction (Agustí et al., 2017). This is only one example of how technical issues during early sample treatment steps can affect the conclusions of the analysis. For this reason, it is our opinion that despite the valuable conclusions, some microbiome analyses based on vPCR have hidden weaknesses.

In the paper by Young et al. (2017), the authors analyzed stool samples that were suspended in PBS, but the pH and the oxygen levels of standard PBS solutions were not optimal for ensuring the viability of the cells in the stool samples. Therefore, the sample handling steps themselves could have affected the viability of some cells in the specimen. For example, only half of the anaerobic microorganisms from the mammalian large bowel survive exposure to O_2_ for 5 min, and this percentage decreases to 3–5% after 20 min of exposure (Brusa et al., 1989). This effect is quite predictable and easy to understand; however, other important aspects, such as centrifugation (Peterson et al., 2012), the time between sample collection and storage **(**Cuthbertson et al., 2014), and freeze/thawing (Cuthbertson, 2015) can also lead to bias in culture-independent analysis.

The authors evaluated the effect of freezing the samples and concluded that freeze/thawing did not change the results for the stool samples. However, factors other than storage were not considered, so it remains unclear whether their conclusions were influenced by additional biases.

The work of Young et al. is valuable, and the conclusions very clearly show the importance of overcoming bias due to DNA from dead cells in a microbial diversity analysis. We suggest that an additional warning about the importance of appropriate sample handling would be helpful to readers who may wish to conduct similar analyses.

## Author contributions

Both authors reviewed the original work and wrote this commentary.

### Conflict of interest statement

The authors declare that the research was conducted in the absence of any commercial or financial relationships that could be construed as a potential conflict of interest.
